# Perfectionism and choice deferral in online shopping: a moderated mediation model of fear of missing out and upward social comparison

**DOI:** 10.3389/fpsyg.2026.1809532

**Published:** 2026-06-03

**Authors:** Yakun Ni, Yuxin Cao, Huiyan Lin, Tengfei Guo, Xiuting Wen

**Affiliations:** 1 School of Psychology and Entrepreneurship Education, Guangdong University of Finance, Guangzhou, China; 2 Faculty of Psychology, Tianjin Normal University, Tianjin, China; 3 School of Educational Science, Guangdong Polytechnic Normal University, Guangzhou, China

**Keywords:** choice deferral, fear of missing out, online shopping, perfectionism, upward social comparison

## Abstract

With the rapid growth of e-commerce, online shopping has become a major channel for consumption. However, the wide range of available options often creates psychological pressure for certain consumer groups. This study examines the mechanisms associated with choice deferral, shifting the focus from external environmental factors to intrinsic personality traits. Based on a sample of 611 participants, we employed a moderated mediation model to explore the relationships among perfectionism, Fear of Missing Out (FoMO), and upward social comparison. The results suggest that: (1) perfectionism is positively associated with choice deferral in online shopping; (2) FoMO functions as a key mediating variable in the relationship between perfectionism and choice deferral; and (3) upward social comparison not only strengthens the relationship between perfectionism and FoMO but is also associated with a stronger indirect relationship between perfectionism and choice deferral. These findings shed light on the psychological antecedents of decision paralysis and provide theoretical insights into personality-related consumer behavior, as well as practical implications for e-commerce platforms seeking to optimize marketing strategies and support consumer self-regulation.

## 1 Introduction

With the advancement of technology and widespread internet access, online shopping has become the primary channel for everyday purchases. This rapid growth has overcome many geographical and age-related barriers of traditional retail. While it delivers unmatched convenience, it has also reshaped digital consumption patterns (Jin, 2024). E-commerce platforms now meet diverse consumer needs, yet they often overwhelm shoppers with vast amounts of information, intensifying decision-making challenges. This information overload is associated with greater difficulty in choosing, poorer shopping experiences, and lower satisfaction, a phenomenon known as choice deferral. Choice deferral occurs when consumers pass up desirable products or promotions due to inaction, often accompanied by negative emotions like disappointment and regret, along with shifts in purchase intentions and behavior. Exploring the drivers and mechanisms of choice deferral in online shopping is thus crucial for enhancing consumer experiences, easing cognitive burdens, boosting decision efficiency and post-purchase satisfaction, and fostering healthier habits (Carmon et al., 2003; Iyengar and Lepper, 2000; Schwartz, 2005).

Prior studies on choice deferral have largely examined contextual factors, such as product information volume and complexity, assortment size and variety, time-limited promotions, price volatility, and information attributes (e.g., Alavi et al., 2015; Dhar, 1997; Huang, 2018). These elements are linked to heightened cognitive load and reduced decision efficiency. Domestic researchers have probed e-commerce specifics like information display formats and social influences (Huang, 2018), but individual psychological traits remain underexplored. Individual differences, however, likely play a major role in shaping choice deferral. Perfectionistic tendencies, in particular, form a stable trait marked by striving for flawless choices and error avoidance. This drives consumers to endlessly seek and compare information, often delaying decisions (Simonson, 1992; Yen and Chuang, 2008). This study thus investigates the link between perfectionistic tendencies and choice deferral in online shopping, including the mechanisms at play.

Perfectionism theory holds that perfectionists collect more information before and during purchases, driven by a quest to maximize outcomes and concerns over overlooking key details or falling short of ideals. These concerns are associated with challenges in timely evaluation of options and gap assessment between expectations and reality, along with elevated error risks. Perfectionists thus experience heightened fear of missing out (FoMO). Elevated FoMO is also linked to anxiety (Milyavskaya et al., 2018), which correlates with deeper information processing in decisions (Xu, 2021)—a resource-intensive process tied to choice deferral (Milgram and Naaman, 1996). We propose that perfectionistic tendencies are associated with choice deferral via FoMO.

Social comparison theory defines upward comparison as selecting superior others as benchmarks to spur self-improvement (Festinger, 1954; Suls and Miller, 1977). Research shows that observing others' superior experiences via upward comparison correlates with intensified FoMO (Reer et al., 2019). Perfectionists, fixated on excellence, may perceive their lives as lacking others' rewards, amplifying feelings of incompleteness (Liu, 2025). They thus scrutinize others' information more closely, linked to heightened FoMO. We argue that upward social comparison strengthens the association between perfectionistic tendencies and FoMO.

**Figure 1:**
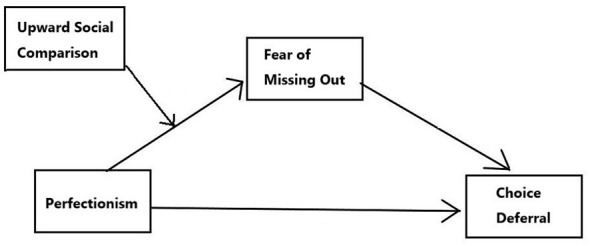
Theoretical model.

In sum, drawing on perfectionism and social comparison theories, this study tests the path from perfectionistic tendencies to choice deferral, mediated by FoMO and moderated by upward social comparison (see Figure 1). Key contributions include: First, shifting focus from external factors like product details and prices to personal traits, broadening antecedents of choice deferral. Second, extending FoMO's role from social media and addiction to online shopping, enriching its theory and illuminating purchase psychology. Third, revealing upward social comparison's moderating effect, with implications for guiding healthy consumption and helping perfectionists refine cognition and habits for better online decisions.

## 2 Theory and hypotheses

### 2.1 Perfectionistic tendencies and choice deferral

The dual-factor model of perfectionism suggests that consumers with perfectionistic tendencies focus on outcomes and utility, emphasizing evaluative aspects (Chang et al., 2021; Slade and Owens, 1995). These traits correlate with sensitivity to decision uncertainties, vulnerability to emotional influences, and reduced decision confidence, all linked to choice deferral (Garg et al., 2017; Hu, 2023). First, perfectionistic tendencies align with decisional procrastination, often tied to fear, anxiety, and low confidence from excessive perfection pursuits, a pattern viewed as a psychological defense (Shi and Yue, 2009; Zhang and Wang, 2014). Second, perfectionists display a maximizing decision style (Andrew et al., 2007), scrutinizing product details and flaws through in-depth analysis and comparison, which extends decision timelines. Compared to satisficers, perfectionists devote more time and effort to option searches (Schwartz, 2009), browse more products (Chowdhury et al., 2009), and engage in broader information seeking (Yang and Chiou, 2010). When perfectionists compare options for ideals, cognitive demands may exceed capacity (Misuraca and Teuscher, 2013), correlating with choice deferral (Chernev et al., 2010; Dhar, 1997).

Hypothesis 1: Consumers' perfectionism correlates positively with their choice deferral.

### 2.2 The mediating effect of FoMO

This study proposes that perfectionistic tendencies relate to choice deferral through FoMO. First, perfectionistic tendencies align with elevated FoMO during consumption. Personality traits influence FoMO development (Blackwell et al., 2017). Perfectionists often show high conscientiousness and neuroticism, both positively linked to FoMO (Blackwell et al., 2017; Chen and Zheng, 2019; Jiang et al., 2020). From a self-evaluation view, Frost et al.'s (1990) core perfectionism features, “concern over mistakes,” “doubts about actions,” and “personal standards,” correlate negatively with self-esteem (Jong, 2002; Kocovski and Endler, 2005). Lower self-esteem, in turn, associates with higher FoMO (Buglass et al., 2016; Chen, 2024).

Second, FoMO correlates with choice deferral. Emotions relate closely to such behavior (Etkin and Ghosh, 2018; Li and Fu, 2006; Simonson, 1992). FoMO reflects anxiety and negative emotions (Milyavskaya et al., 2018), linking to greater choice deferral tendencies (Li and Xie, 2012). In decisions, reducing negative emotions serves as a key aim, with choice deferral associating with emotion management amid FoMO (Luce, 1998).

FoMO differs fundamentally from regret and maximizing. Regret looks backward at past choices, while FoMO involves forward-looking anxiety over missed opportunities (Zeelenberg and Pieters, 2007; Przybylski et al., 2013; Rowson, 2026). Maximizing is a cognitive style (Schwartz et al., 2002), whereas FoMO is emotional; maximizing can precede FoMO (Doǧan et al., 2025). Uncertainty avoidance links to decision avoidance (Hofstede, 2001), but high FoMO prompts active information seeking and decision delay (Flecha Ortiz et al., 2023).

Perfectionists thus experience self-doubt and frustration in choices, tied to error concerns, action doubts, and high standards. These link to lower self-esteem and higher FoMO. Amid FoMO-related pressure, choice deferral serves as a coping pattern.

Hypothesis 2: FoMO mediates the relationship between perfectionism and choice deferral.

### 2.3 The moderating effect of upward social comparison

Upward social comparison involves individuals benchmarking against superior performers (Wheeler, 1966). Moderate levels aid self-understanding, ability evaluation, and improvement goals by highlighting gaps, associating with self-enhancement (Wheeler, 1966). Excessive levels, however, correlate with negative effects: overly positive views of others' lives foster inferiority, negative emotions, and reduced self-esteem (Fardouly et al., 2015; Vogel et al., 2014). Lower self-esteem links to higher FoMO.

For perfectionists, frequent upward comparison introduces external standards, correlating with insecurity, failure concerns, and elevated FoMO. Infrequent comparison shifts focus to internal standards, where frustration arises from unmet personal goals rather than relative inferiority, associating with lower FoMO.

Hypothesis 3: Upward social comparison moderates positively the relationship between perfectionistic tendencies and FoMO. The relationship strengthens at high levels of upward social comparison and weakens at low levels.

Building on perfectionism and social comparison theories, plus Hypotheses 1–3, upward social comparison moderates the indirect link between perfectionistic tendencies and choice deferral via FoMO. Perfectionists engaging in frequent upward comparison note personal shortcomings and missed alternatives, linking to stronger FoMO and choice deferral. Low-comparison perfectionists rely on internal standards, showing weaker indirect links via FoMO.

Hypothesis 4: Upward social comparison moderates the indirect relationship between perfectionism and choice deferral through FoMO. The indirect relationship strengthens at high levels of upward social comparison compared to low levels.

## 3 Research methods

### 3.1 Participants and procedure

This study used convenience sampling to survey 673 participants across age groups in Guangzhou, Nanjing, Harbin, and Gansu. Data collection occurred via an online questionnaire with three parts: instructions, demographics, and variable scales. Distribution happened through the Wenjuanxing mini-program. Participants received full details on study goals and requirements; involvement remained voluntary. Anonymity assurances encouraged accurate, complete responses. The lead researcher screened replies, excluding invalid cases (e.g., patterned answers or completion under 100 s). This yielded 611 valid responses: 263 males (43.0%) and 348 females (57.0%). Ages broke down as 16–20 (3.1%), 21–25 (44.2%), 26–30 (33.2%), 31–35 (11.9%), and over 35 (7.5%). Education levels included associate or below (8.0%), bachelor's (67.1%), and master's or higher (24.9%). Occupations comprised enterprise employees (37.8%), students (30.4%), educators (12.3%), public servants (9.5%), self-employed (8.0%), and others (2.0%). Monthly household income ranged from ≤ 3,000 yuan (19.8%), 3,001–6,000 (39.4%), 6,001–10,000 (26.8%), 10,001–15,000 (9.3%), to >15,000 (4.6%). Online shopping frequency showed 27.7% monthly or more, 42.9% weekly or more, 14.7% for nearly all necessities, 10.8% a few times yearly, and a small group never.

### 3.2 Measurement instruments

All scales used a 5-point Likert format (1 = strongly disagree; 5 = strongly agree).

#### 3.2.1 Perfectionism

The study adapted Frost et al.'s (1990) Frost Multidimensional Perfectionism Scale (FMPS), using Zi's (2007) Chinese revision. It includes 27 items, such as “My parents set very high standards for me.” Higher scores reflect stronger perfectionistic tendencies. Cronbach's α was 0.97.

#### 3.2.2 Upward social comparison

The upward social comparison subscale came from Gibbons and Buunk (1999), revised by Bai et al., 2013. It has six items, such as “When things go badly, I often think about people who are doing better than I am.” Higher scores indicate greater upward comparison tendencies. Cronbach's α was 0.92.

#### 3.2.3 Choice deferral

Walsh et al. (2007) choice deferral scale, revised by Hua and Yang (2023), measured this construct with three items, such as “When shopping online, I sometimes choose to postpone my purchase plans.” Higher scores signal stronger deferral tendencies. Cronbach's α was 0.85.

#### 3.2.4 FoMO

Przybylski et al.'s (2013) FoMO Scale (FoMOs), revised by Li Qi, covers two dimensions (FoMO on Information; Situational FoMO) across eight items. An example: “I am afraid that my friends have more exciting experiences and achievements than I do.” Higher scores denote greater FoMO. Cronbach's α was 0.92 overall (0.88 for Information; 0.84 for Situational).

### 3.3 Analytical strategy

Harman's single-factor test (Zhou and Long, 2004) checked for common method bias. Descriptive statistics and Pearson correlations examined variable relations. PROCESS macro Models 4 and 7 in SPSS tested mediation and moderation, respectively.

## 4 Results

### 4.1 Test for common method bias

Harman's single-factor test (Zhou and Long, 2004) assessed common method bias. Principal components analysis with varimax rotation showed the largest factor explaining 32.81% of variance—below the 40% threshold. This indicates no substantial common method effects.

### 4.2 Normality test

Shapiro-Wilk tests evaluated core variables: perfectionism (*W* = 0.952, *p* < 0.001), upward social comparison (*W* = 0.965, *p* < 0.001), FoMO (*W* = 0.976, *p* < 0.001), and choice deferral (*W* = 0.956, *p* < 0.001). All deviated from normality. With *N* = 611, however, this test proves sensitive to minor deviations (Kim, 2013; Shatz, 2024). Skewness ranged from −0.263 to 0.032 (absolute < 2), and kurtosis from −1.047 to −0.810 (absolute < 7; see Table 1; Curran et al., 1996). Data showed approximate normality. Parametric methods suit mild non-normality (Bishara and Hittner, 2012), so Pearson correlations and regressions proceeded.

**Table 1:** Normality test results for key variables (*N* = 611).

Variable	Kolmogorov-Smirnov		Shapiro-Wilk	
	Statistic	*p*	Statistic	*p*
Perfectionism	0.071	<0.001	0.952	<0.001
Upward social comparison	0.082	<0.001	0.965	<0.001
FOMO	0.049	0.002	0.976	<0.001
Choice deferral	0.117	<0.001	0.956	<0.001

### 4.3 Multicollinearity test

Variance inflation factor (*VIF*) and tolerance checked multicollinearity among predictors (Field, 2024; Tabachnick and Fidell, 2019). Perfectionism scored *VIF* = 1.50, tolerance = 0.67; FoMO, *VIF* = 1.45, tolerance = 0.69; upward social comparison, *VIF* = 1.60, tolerance = 0.63 (Table 2). All VIFs stayed below 5 and tolerances above 0.2, confirming no multicollinearity issues for regression.

**Table 2:** Collinearity diagnostics for the predictor variables (*N* = 611).

Variable	Tolerance	VIF
Perfectionism	0.67	1.5
Upward social comparison	0.63	1.6
FOMO	0.69	1.45

*VIF* = variance inflation factor.

### 4.4 Correlation analysis

Table 3 presents Pearson correlations. Perfectionism correlated positively with upward social comparison (*r* = 0.534, *p* < 0.001), FoMO (*r* = 0.462, *p* < 0.001), and choice deferral (*r* = 0.421, *p* < 0.001). Upward social comparison linked positively to FoMO (*r* = 0.512, *p* < 0.001) and choice deferral (*r* = 0.454, *p* < 0.001). FoMO and choice deferral showed a positive correlation (*r* = 0.350, *p* < 0.001).

**Table 3:** Means, standard deviations, and correlation analysis results for each variable (*N* = 611).

Variable	M	SD	1	2	3	4
1. Perfectionism	3.29	0.92	1			
2. Upward social comparison	3.36	0.97	0.534^***^	1		
3. FoMO	3.37	0.91	0.462^***^	0.512^***^	1	
4. Choice deferral	3.45	1.00	0.421^***^	0.454^***^	0.350^***^	1

^***^p < 0.001.

### 4.5 Hypothesis testing

H1: Perfectionism relates positively to choice deferral. In Table 4 (Model 3), after controls, perfectionism showed a significant positive beta (β = 0.05, *SE* = 0.004, *p* < 0.001), with *R*^2^ = 0.45 and Δ*R*^2^ = 0.21 (*p* < 0.001). H1 holds.

**Table 4:** Regression results (*N* = 611).

Variable	FoMO		choice deferral	
	M1	M2	M3	M4
Controls				
Gender	−0.96^***^(0.53)	−0.87(0.49)	0.16(0.22)	0.25(0.22)
Age	0.65(0.34)	0.49(0.31)	−0.25(0.14)	−0.32(0.14)
Culture	1.12^**^(0.41)	0.96^*^(0.37)	−0.0748	−0.55^**^(0.17)
Occupation	−0.12(0.23)	−0.05(0.21)	−0.0171	−0.18(0.09)
Income	−0.80^**^(0.27)	−0.072^**^(0.25)	0.21(0.11)	0.29^*^(0.11)
Online shopping Frequency	−0.23(0.26)	−0.14(0.24)	−0.03(0.11)	−0.006(0.11)
Predictors				
Perfectionism	0.13^***^(0.01)	0.06^***^(0.01)	0.05^***^(0.004)	0.04^***^(0.005)
Mediator				
FoMO				0.10^***^(0.02)
Moderator				
Upward social comparison		0.44^***^(0.05)		
Perfectionism^*^Upward social comparison		0.01^***^(0.002)		
*R^2^*	0.49^***^	0.60^***^	0.45^***^	0.50^***^
*ΔR^2^*	0.24^***^	0.37^***^	0.21^***^	0.25^***^
*F*	27.61^***^	38.47^***^	22.25^**^	24.57^***^

^*^ *p* < 0.05;

^**^ *p* < 0.01;

^***^ *p* < 0.001.

H2: FoMO mediates between perfectionism and choice deferral. Table 4 (Models 1 and 4) shows perfectionism relating positively to FoMO (β = 0.13, *SE* = 0.01, *p* < 0.001; *R*^2^ = 0.49, Δ*R*^2^ = 0.24, *p* < 0.001). FoMO related positively to choice deferral (β = 0.10, *SE* = 0.02, *p* < 0.001). The direct path remained significant (β = 0.04, *SE* = 0.005, *p* < 0.001), with *R*^2^ = 0.50 and Δ*R*^2^ = 0.25 (*p* < 0.001). Table 5 confirms partial mediation (effect = 0.25). H2 holds.

**Table 5:** Bootstrap test of the mediating effect (Bootstrapping = 5000).

Path	Effect	SE	LLCI	ULCI
Total effect	0.052	0.004	0.04	0.06
Direct effect	0.039	0.003	0.01	0.02
Mediating effect (Perfectionism → FoMO → choice deferral)	0.013	0.005	0.00	0.03

**Figure 2:**
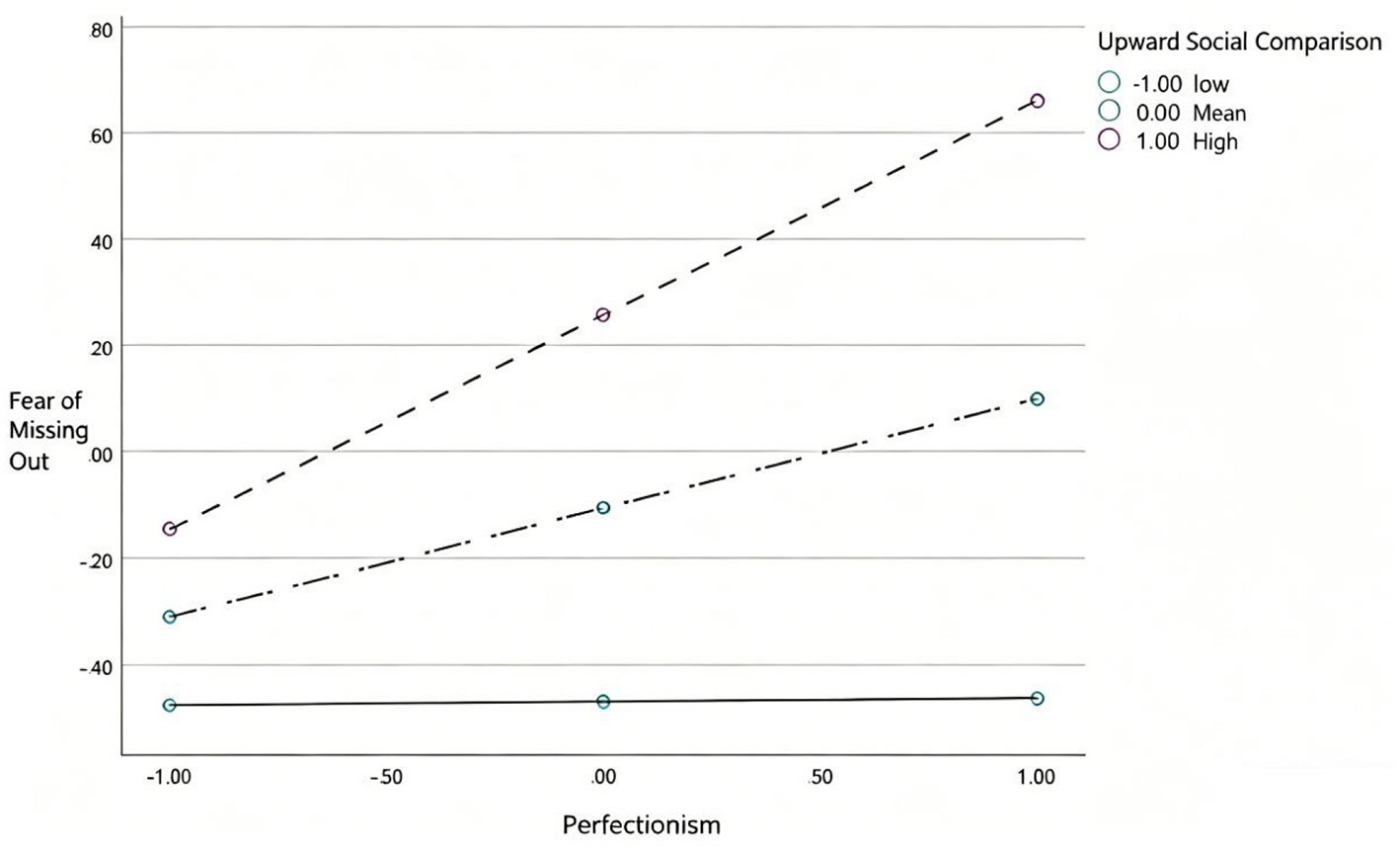
Simple slope test of the moderating effect.

H3: Upward social comparison moderates positively between perfectionism and FoMO. PROCESS Model 7 tested this. Table 4 (Model 2) shows the interaction term relating positively to FoMO (β = 0.01, *SE* = 0.002, *p* < 0.001; *R*^2^ = 0.60, Δ*R*^2^ = 0.37, *p* < 0.001). Moderated mediation index = 0.001, 95% CI [0.0004, 0.0015] excluding zero. Figure 2 illustrates simple slopes: at low upward social comparison (−1 SD), β = 0.002 (*SE* = 0.018, *t* = 0.10, *p* = 0.92); at high (+1 SD), β = 0.12 (*SE* = 0.01, *t* = 8.58, *p* < 0.001). H3 holds.

H4: Upward social comparison moderates the indirect path from perfectionism to choice deferral via FoMO. PROCESS Model 7 results (Table 6) show: at low levels (−1 SD), indirect effect = 0.0002, 95% CI [−0.004, 0.004]; at high (+1 SD), indirect effect = 0.011, 95% CI [0.007, 0.016]. The moderation strengthens the indirect path at high levels. H4 holds.

**Table 6:** Test of the moderated mediating effect.

Comparison level	Perfectionism→**FoMO**→choice deferral			
	β	SE	LLCI	ULCI
Low level of upward social comparison (M – 1 SD)	0.0002	0.002	−0.004	0.004
Mean	0.006	0.002	0.003	0.009
High level of upward social comparison (M + 1 SD)	0.011	0.003	0.007	0.016

**Figure 3:**
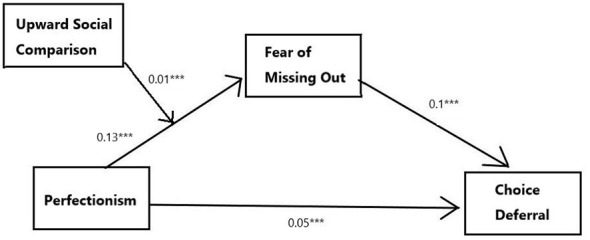
Moderated mediation model.

Figure 3 depicts the full moderated mediation model.

## 5 Discussion

### 5.1 Research conclusions

This study draws on perfectionism and social comparison theories to explore links among perfectionistic tendencies, FoMO, upward social comparison, and choice deferral in online shopping. Results show: perfectionistic tendencies correlate positively with choice deferral; FoMO mediates between perfectionistic tendencies and choice deferral; upward social comparison moderates positively between perfectionistic tendencies and FoMO, with stronger links at high levels.

### 5.2 Theoretical significance

First, this study shifts focus from external factors (e.g., decision environments) to personal traits in choice deferral research, broadening antecedents. Prior work emphasized product details (Huang, 2018), time pressure (Dhar and Nowlis, 1999; Zhu et al., 2019), and situational variables. While emotions received some attention (Simonson, 1992; Yen and Chuang, 2008), stable traits like perfectionism remain underexplored in online contexts (Shi and Yue, 2009; Zhang and Wang, 2014). Findings link perfectionistic tendencies to choice deferral, consistent with associations between procrastination, perfectionism, fear, and anxiety (Shi and Yue, 2009; Zhang and Wang, 2014). Perfectionists set high standards for recognition and optimal outcomes (Chang et al., 2021), correlating with decision pressure and patterns of postponement (Garg et al., 2017).

Second, examining FoMO's mediating role clarifies choice deferral processes. Online shopping limits direct product access (Zhang, 2011), yet perfectionists prioritize expected outcomes (Chang et al., 2021). Information gaps associate with uncertainty and risk perceptions (Wu, 2008), linking to insecurity, anxiety, and FoMO. Unlike uncertainty avoidance (Hofstede, 2001), regret (Zeelenberg and Pieters, 2007), or maximizing (Schwartz et al., 2002), FoMO motivates extensive searches, correlating with longer decisions and deferral.

Third, social comparison theory frames upward social comparison's moderating role, extending links from perfectionism to decisions. Individuals compare against others absent objective standards (Festinger, 1954). High upward comparison among perfectionists shifts to external benchmarks, associating with pressure, low self-esteem, and FoMO sensitivity. Success observations heighten missed-opportunity concerns (Pan et al., 2023).

### 5.3 Practical significance

This study highlights psychological patterns in perfectionists' online decisions, offering guidance for consumers and platforms.

First, retailers targeting perfectionists should use concise, focused product descriptions and limit trivial details to curb overanalysis. Simplified options or bundles aid quicker selections amid abundant choices.

Second, platforms can add filters to search results, reducing homogeneous item overload. Refining reviews may temper upward comparison, linking to lower FoMO and deferral.

Third, perfectionist consumers benefit from pre-shopping goal-setting and limiting non-essential info exposure, associating with reduced anxiety and deferral risks.

### 5.4 Research limitations and future directions

This study has limitations that suggest avenues ahead.

First, its cross-sectional design limits causal claims. Longitudinal designs could explore temporal patterns.

Second, participants centered on ages 21–30; older groups, including less frequent online shoppers, remain unexamined. Broader age samples would test generalizability.

Third, self-reports risk bias from disclosure reluctance or poor introspection; online formats add attention concerns. Future work could use behavioral observations, implicit measures, or simulated shopping experiments.

Fourth, FoMO theory lacks depth on perfectionism-decision links. Further reviews, qualitative studies, and experiments would refine these associations.

Finally, cultural factors (e.g., norms, individualism-collectivism, upbringing) went unexamined. Including them could reveal contextual influences on perfectionists' decisions.

## 6 Conclusion

This study links perfectionistic tendencies, FoMO, upward social comparison, and choice deferral in online shopping. Perfectionistic tendencies associate with choice deferral via FoMO, moderated by upward social comparison—stronger at high levels, where external focus amplifies FoMO. Lower upward comparison ties to less external attention, FoMO, and deferral. As the first to place FoMO in consumer decisions, it clarifies perfectionism-deferral patterns with strong theoretical and practical value.

## Data Availability

The raw data supporting the conclusions of this article will be made available by the authors, without undue reservation.
